# Polysomnography evaluation and swallowing endoscopy of patients with Pierre Robin Sequence

**DOI:** 10.1016/S1808-8694(15)30549-8

**Published:** 2015-10-19

**Authors:** Carlos Diógenes Pinheiro Neto, Nivaldo Alonso, Luiz Ubirajara Sennes, Dov Charles Goldenberg, Patrícia de Paula Santoro

**Affiliations:** 1 Intern in Skull-Maxillofacial Surgery and Skull Base Surgery - Department of Ophthalmology and Otorhinolaryngology of the University of São Paulo Medical School - FMUSP; 2 FMUSP. Head of the Skull-Maxillofacial Group - FMUSP; 3 Otorhinolaryngology - FMUSP. Head of the Skull-Maxillofacial Surgery Group FMUSP; 4 Medicine - FMUSP. Assistant Physician - Plastic Surgery Department - University of São Paulo Medical School Hospital (HCFMUSP); 5 Medicine - FMUSP. Assistant ENT and Head of the Dysphagia Ward HC-FMUSP. Hospital das Clínicas da Faculdade de Medicina da USP

**Keywords:** craniofacial abnormalities, deglutition, endoscopy, polysomnography, pierre robin syndrome

## Abstract

The Pierre Robin sequence is characterized by micrognathia, glossoptosis and upper airway obstruction. Symptom severity varies, and this makes the treatment of these patients a true challenge.

**Aim:**

to identify the presence of sleep hypopnea-apnea in patients with Pierre-Robin sequence.

**Materials and Methods:**

retrospective study in which we assessed 14 children with Pierre-Robin sequence, eight girls. The children were submitted to swallowing video-endoscopy study and polysomnography.

**Results:**

eight patients were included in this study. Six had normal polysomnography and only one patient had mild central hypopnea-apnea. Swallowing video-endoscopy was normal in five patients and moderate dysphagia was detected in three patients, who were then submitted to gastrostomy. Mandible distraction was carried out in four patients who were also submitted to tracheostomy during the same procedure.

**Conclusions:**

dysphagia was more prevalent than sleep apnea. Swallowing video-endoscopy proved to be a dynamic test and one able to detect feeding disorders in patients with Pierre Robin sequence.

## INTRODUCTION

In 1934, the French physician Pierre Robin described a congenital anomaly characterized by micrognathia, glossoptosis and upper airway obstruction. A palatine fissure is frequently associated with this sequence^1^. It is estimated that the Pierre Robin sequence affects 1 child for every 8,500 births^2^ and it can occur alone or as part of some syndrome, such as the Treacher Collins, Stickler, bilateral craniofacial microsomia and the fetal alcoholic syndrome^3^.

Micrognathia is the basic anatomical component of the Pierre Robin sequence. Mandible size reduction is responsible for the retroposition of the supra-hyoid muscle which reflects on a reduction in oropharyngeal capacity and glossoptosis^1^. Respiratory failure can manifest in different levels, and sometimes a tracheostomy is necessary^4^.

A palatine fissure happens in 90% of the cases. Of these, 70% are broad and complete fissures, while 30% are narrow, complete or incomplete^5^. Posterior and superior tongue positioning during the embryonic period prevents the fusion of palatine leaflets, which happens between the eighth and the tenth gestational week^6^.

Other alterations that children with Pierre Robin sequence may present are feeding difficulties. These difficulties are common and, in general, stem from swallowing alterations resulting in malnutrition and deterioration of the child's clinical condition. In cases of severe dysphagia, patients may present with aspiration pneumonia, thus considerably increasing the morbid-mortality of these patients^7^.

The severity of clinical manifestations in patients with Pierre Robin sequence varies substantially. Caouette-Laberge et al. Proposed a classification in three categories: proper breathing in ventral decubitus and proper feeding (category I), proper breathing in ventral decubitus and feeding difficulties (category II), respiratory failure with need for intervention and feeding difficulties with a need for gavage (category III)^7^.

Because of the major heterogeneity of the clinical manifestations and its variable severity spectrum, these patients are often challenging in their treatment. There is no consensus in the literature as to the best treatment option for these patients^8^. There are very few studies which assessed the respiratory pattern during sleep in these patients by means of a polysomnography (PSG). So far, we have not found any paper published assessing the presence of dysphagia/aspiration through swallowing videoendoscopy (SVE).

The present paper aims at detecting the presence of sleep obstructive apnea-hypopnea by means of polysomnography and assess the presence of swallowing alterations in patients with Pierre Robin Syndrome by means of SVE.

## METHODS

Fourteen children were diagnosed as having Pierre Robin sequence and were followed up by the Skull-Maxillofacial surgery group between January of 2006 and April of 2008. The diagnosis was established through the clinical evaluation and the finding of the triad: micrognathia, glossoptosis and some degree of respiratory difficulty^1^. We included in the study only those children assessed by means of swallowing video-endoscopy and polysomnography or acute respiratory failure in need of urgent intervention. Thus, we excluded all the children who were not submitted to swallowing video-endoscopy. All the patients who were not submitted to polysomnography were also excluded, except for those who had acute respiratory failure and required urgent care. Syndromic patients with Pierre Robin were also taken off the sample. A total of 8 children were included in this study. This paper was approved by the Ethics Committee of the institution under protocol # 0552/08.

**Figure 1:**
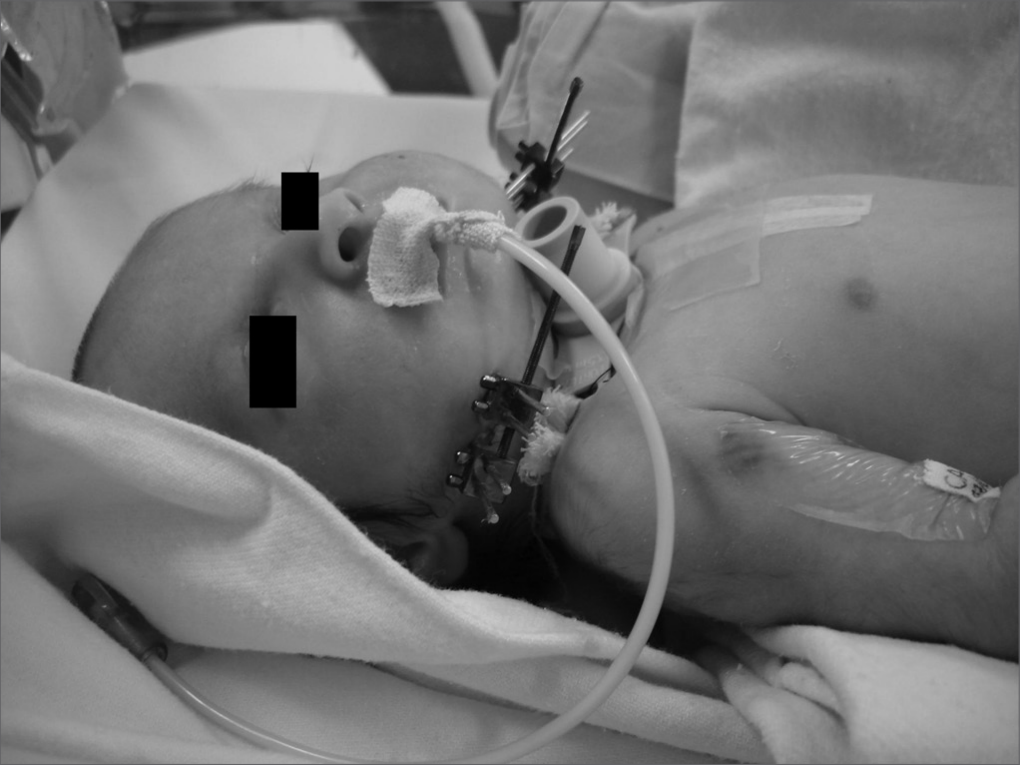
Newborn with Pierre Robin sequence at four days of life: bilateral mandibular osteotomy and tracheostomy with the placement of an external distracter. Notice the presence of an orogastric tube as an alternative source of feeding.

The diagnosis of sleep obstructive apnea-hypopnea was done by means of polysomnography (PSG). An apnea-hypopnea index (AHI) below 1 event per hour was considered normal. Seven children were submitted to the test. PSG was carried out always with the same intent. One of the children had acute respiratory failure immediately after birth, and was submitted to tracheostomy and mandible distraction within a few days of life. It was not possible to perform PSG before the procedure (Figure 1).

Besides being used for diagnostic purposes, PSG was also used to quantify the apnea severity of the patients. Mild apnea was considered when there was and AHI equal to or higher than 1 event per hour up to an index lower than 5 events per hour. An AHI higher than 5 events per hour up to an index lower than or equal to 10 events per hour characterizes apnea as moderate. Severe apnea was considered in the presence of an AHI greater than 10 events per hour^9^.

All the children were submitted to swallowing videoendoscopy (SVE) for swallowing structural and functional evaluation. The tests were always performed by the same physician. For all the tests we used an Olympus® 4.2mm ENF type 10 flexible nasofibroscope. We offered food bolus dyed with aniline blue (edible dye) in progressive consistencies and quantities. The patients were classified as having normal swallowing; mild, moderate or severe dysphagia. Severity diagnosis and quantification were established by the examining physician based on criteria such as: clearing of food bolus after each swallowing, food penetration in the supraglottis and aspiration, the presence of food below the vocal folds (severe dysphagia), amongst other criteria^10^.

## RESULTS

Regarding patient distribution by gender, of the eight patients, six were females (75%). All the patients had their palatine clefts surgically corrected.

Mandible osteogenic distraction was carried out in 50% of the patients, who were also submitted to tracheostomy in the same surgical act. The respiratory distress of the remaining 50% of the patients was treated conservatively, by means of positional therapy, without the need of any intervention to control the upper airways.

In regards of the polysomnography, six patients had normal PSG and only one patient had mild central hypopnea-apnea. One of the patients was submitted to tracheostomy and mandible osteogenic distraction at four days of life because of respiratory failure, and preoperative PSG was not done.

As far as SVE results are concerned, it proved normal in five patients and moderate dysphagia was detected in three patients who were submitted to gastrostomy. Except for the newborn submitted to tracheostomy and mandible osteogenic distraction at four days of life, SVE was done before treating the other patients.

Results are summarized on Table 1.

**Table 1:** Patients seen by the Skull-Maxillofacial Surgery Group.

Pct	Gender	Palatine cleft	Distracter	PSG^1^	SVE^2^	Tracheo	Gastrostomy
1	F	Yes	No	Normal	Normal	No	No
2	F	Yes	No	Normal	Normal	No	No
3	F	Yes	Yes	Normal	Moderate dysphagia	Yes	Yes
4	F	Yes	Yes	Normal	Moderate dysphagia	Yes	Yes
5	M	Yes	No	Mild apnea	Normal	No	No
6	F	Yes	Yes	Normal	Normal	Yes	No
7	M	Yes	No	Normal	Normal	No	No
8^3^	F	Yes	Yes	Not done	Moderate dysphagia	Yes	Yes

1 PSG = Polysomnography

2 SVE = Swallowing Video-Endoscopy

3 Patient did not undergo PSG because she was submitted to tracheostomy at four days of life because of respiratory failure, therefore no preop exam was carried out.

## DISCUSSION

Meyer and co-workers studied 53 non-syndromic children with Pierre Robin (PR) sequence and reported a prevalence of 64.2% in females^8^. Schaefer et al. studied 21 patients with the PR sequence alone and reported 66.7% prevalence in females^11^. We found 75% of female patients. Therefore, both our results and those found in the literature showed a larger prevalence of non-syndromic Pierre Robin sequence in females when compared to their male counterparts.

Glossoptosis, with a consequent upper airway obstruction is a prominent characteristic of the Pierre Robin sequence. Its degree of respiratory distress is variable. Conservative treatment of positioning therapy can be indicated. The patient is placed lying on his belly so as to have the tongue more anteriorly positioned. Conservative treatment gains at not being invasive and having low morbidity^11^.

Some studies show high success rates in the treatment of these patients with positional therapy^11^, ^12^, ^13^, ^14^. Meyer et al., in their series with 74 children reported that 49% did not require intervention for airway control or were successfully treated by means of posture^8^. These data are similar to the ones found in our series – 50% of the patients were treated this way.

In more severe cases, conservative treatment is insuficient^15^. Treatment can be carried out by using a nasopharyngeal tube, glossopexy and, more recently, mandible osteogenic distraction.

Meyer et al. reported that 32% of the children required surgical intervention to control respiratory symptoms; of these, 75% were submitted only to mandible osteogenic distraction, 4% to tracheostomy alone and 21% were submitted to tracheostomy followed by mandible osteogenic distraction. Seventy-five per cent of the patients treated surgically were initially submitted to non-surgical interventions, such as the placement of a nasopharyngeal tube and/or orotracheal intubation^8^. In our study, half of the patients were treated with mandible osteogenic distraction. Tracheostomy was done during the distraction surgery in all these children. Besides tracheostomy, the palatine cleft was also closed. After the distraction, the tracheostomy cannula was removed in all the patients. Only one patient treated surgically was submitted to a nasopharyngeal tube before the procedure.

Monastério et al. evaluated 18 patients through polysomnography. They reported a mean apnea/hypopnea index (AHI) of 18.3/h which is considered severe apnea in the pediatric population. In our study, there was only one patient with altered AHI. This patient's PSG showed mild central apnea. This result discrepancy showed the variability we can see in the airway obstruction of these patients. In Monastério's series, all the children had cyanosis during feeding. Cyanosis was reported in only one patient of our study.

Sleep obstructive apnea pathophysiology is still controversial^16^. As we can see, our study did not detect any sleep obstructive apnea in the 7 patients submitted to PSG. Only one patient had acute respiratory failure and, therefore, overt apnea even when awake. Since all the children had retrognathia, these data challenge the real importance of the facial bone framework in creating sleep obstructive apnea. Muscles and soft tissue seem to have a more prominent function in maintaining the upper airways patent during sleep.

In his study, Monastério et al. assessed the children using swallowing video-fluoroscopy. All the patients had some degree of tongue mobility impairment, 66.6% had contrast penetrating their laryngeal vestibule and 50% had residual material in their pharyngeal recesses1. Meyer et al. reported that 50% required intervention for an alternative feeding pathway. Of the latter, 51% received a nasogastric tube, 19% were submitted to gastrostomy and 30% to gastrostomy after the nasogastric tube placement^8^. In our study, all the children were submitted to swallowing videoendoscopy, therefore being the first study reported in the literature to discuss this test in patients with the Pierre Robin sequence. Of the eight children studied, 37.6% had moderate dysphagia characterized by the food bolus penetrating the laryngeal vestibule region. These patients were submitted to gastrostomy as an alternative path to feeding.

It is important to stress that, in our series; all the patients who had swallowing alteration diagnosed by SVE were submitted to gastrostomy, mandible osteogenic distraction and tracheostomy. Having food bolus penetrate the laryngeal vestibule can represent a factor of worse prognosis and an indication for the need of a more aggressive treatment. Swallowing videoendoscopy has the main advantage of not exposing the patient to ionizing radiation, when compared to the video-swallowgram. It is a not very invasive procedure and has excellent sensitivity and specificity^10^.

## CONCLUSIONS

Sleep obstructive apnea was less prevalent than dysphagia. SVE proved to be a dynamic and efficient test to detect feeding disorders in patients with Pierre Robin sequence. Further studies, with more patients, are necessary in order to establish a correlation between the SVE findings and the type of treatment.
